# Steatocystoma Simplex of the Orbit: Expanding the Differential for Pediatric Eye Masses

**DOI:** 10.7759/cureus.110446

**Published:** 2026-06-08

**Authors:** Alisa Vidwans, Tejasvi Paturu, Syeda Sumara Taranum Basith

**Affiliations:** 1 Ophthalmology, University of South Florida Morsani College of Medicine, Tampa, USA

**Keywords:** dermoid cyst mimic, histopathological diagnosis, orbital mass, pediatric ophthalmology, steatocystoma simplex

## Abstract

Steatocystoma simplex (SS) is a rare cutaneous disorder that often presents as a single benign cyst, most commonly in adults. Due to overlapping clinical features, SS is often mistaken for dermoid cysts until histopathological evaluation is performed and a diagnosis is established. We present a case of a presumed orbital dermoid cyst in a pediatric patient that was ultimately identified as SS, suggesting a possible congenital origin.

A 20-month-old boy presented with a painless, mobile mass of the left upper eyelid that was first noted after minor trauma. Imaging with ultrasound and computed tomography demonstrated a well-circumscribed cystic lesion, most consistent with a dermoid cyst. Anterior orbitotomy with excision of the mass and subsequent histopathological evaluation yielded a squamous epithelial-lined cyst with associated sebaceous lobules and an eosinophilic cuticle consistent with SS. The postoperative course was uncomplicated, and no recurrence has been observed.

SS of the orbit is rare and resembles other common cystic lesions; therefore, diagnosis relies on histopathological evaluation following surgical excision. Literature is limited, as only two other pediatric patients have been reported to have a similar presentation, both without associated systemic findings or family history. This case highlights the importance of including SS in the differential diagnosis of pediatric orbital masses. Increased recognition of this entity may improve diagnostic accuracy and guide appropriate management of orbital lesions in children.

## Introduction

Steatocystoma simplex (SS) is a rare cutaneous disorder characterized by the development of a singular benign squamous epithelial-lined cyst with sebaceous glands and a corrugated eosinophilic lining [1,2]. Because these lesions typically present as well-circumscribed cystic nodules, their appearance is often indistinguishable from other cystic lesions of the periorbital area [3]. While SS has primarily been observed in adults, in a recent report, two cases of facial SS lesions were described in children under the age of two years, suggesting that SS may arise congenitally [4].

This report covers a rare variant of SS, SS of the orbit. A review of the literature yielded no systematic reviews or clinical trials on SS of the orbit; however, two pediatric [4] and six adult (23-68 years) [3,5-10] case reports were identified. None of the patients had a personal or family history of similar lesions. In seven of the eight cases, the lesions were asymptomatic except for some swelling [5-10], while in one case, the patient experienced ptosis in the affected eye [10].

Common differentials before histologic diagnosis included dermoid cyst, epidermoid cyst, and chalazion. All cases described in the literature were successfully treated with surgical excision and diagnosed histologically [3-10]. A review of reported cases demonstrated a female predominance, with lesions affecting the ocular caruncle most frequently, followed by the eyelid and eyebrow. Cases spanned a wide age range, from infancy through adulthood.

Due to the overlapping clinical and radiographic features, SS is often misdiagnosed as more common lesions, such as epidermoid cysts or dermoid cysts. Because the definitive treatment for SS is surgical removal, proper diagnosis has implications for treatment and surgical planning.

## Case presentation

**Figure 1:**
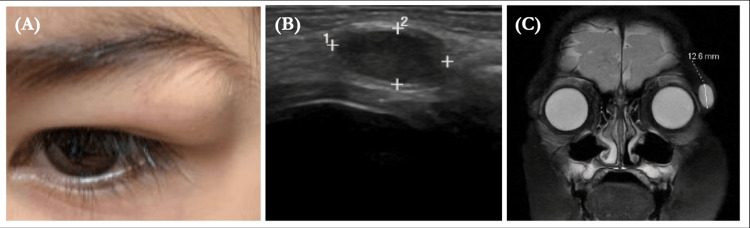
Pediatric patient with a well-defined mass on his left upper eyelid. Panel A shows the gross appearance of the mass at the patient’s initial ophthalmology appointment, panel B the ultrasound, and panel C the computed tomography (CT) scan. All images in this panel are original.

A healthy 20-month-old full-term boy was referred to the eye clinic for a left upper eyelid mass. The swelling on the left upper eyelid was noticed by his mother following trauma to his left eye when he was six months old. She noted that the mass swelled when the patient was agitated. She denied any other lesions in the child and denied family history of similar skin lesions. On exam, the mass was constant, firm, and freely mobile with no change in color (Figure 1A). Initial providers expressed a concern for a left eye hematoma/sebaceous cyst and ordered an orbital ultrasound (Figure 1B) and CT scan (Figure 1C), which demonstrated a cystic hypoechoic mass above the left eye. The patient had age-appropriate hyperopia of both eyes, not requiring treatment. The systemic exam was noncontributory. 

**Figure 2:**
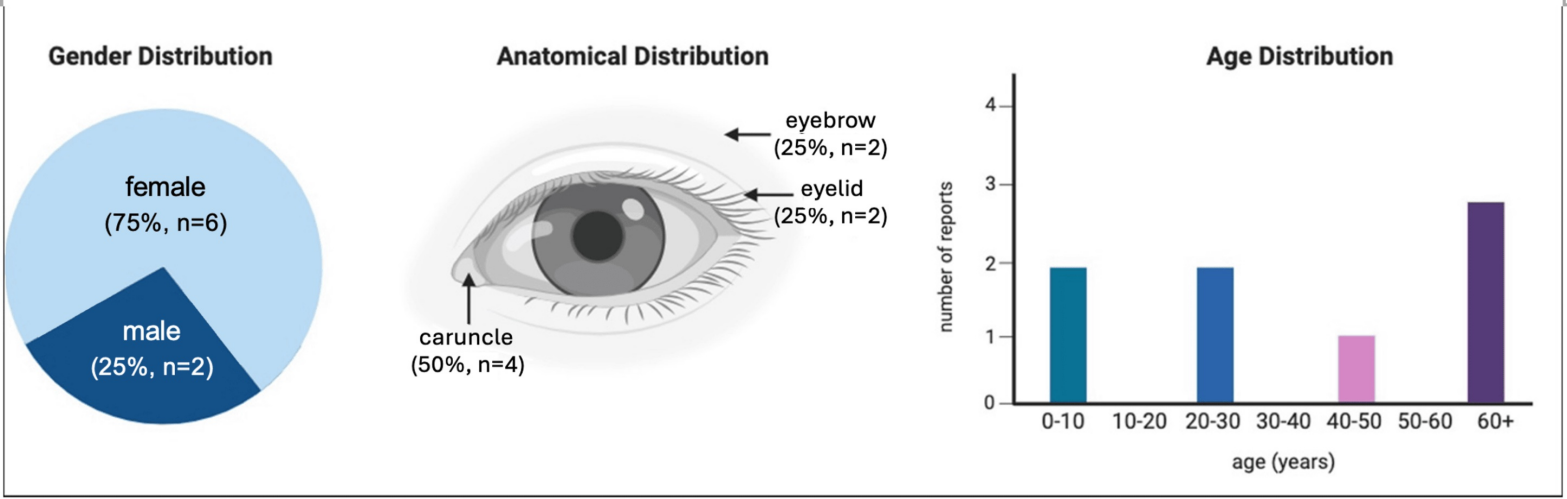
The gender, anatomical, and age distributions of reported cases of orbital steatocystoma simplex. Data compiled from previously published case reports [3-10], and the image was created by the authors using BioRender. No artificial intelligence (AI) tools were used.

Differential diagnoses included dermoid cyst, epidermoid cyst, and hemangioma/lymphangioma. The gender, anatomical, and age distributions of SS described in previous case studies are summarized in Figure 2. Clinical exam and imaging results were most consistent with a dermoid cyst, and surgical excision was planned. A left eye anterior orbitotomy with excision of dermoid was performed under general endotracheal anesthesia. After preparing and draping the eye under sterile precautions, the cornea was protected with a scleral shell in the left eye. A curvilinear incision was created over the cyst with a #15 blade. Hemostasis was achieved, and the cyst was excised using blunt and sharp dissection in the usual surgical manner *in toto* and sent for histopathological evaluation. The excised tissue measured 1.0 x 0.7 x 0.15 cm and was fixed and routinely processed.

**Figure 3:**
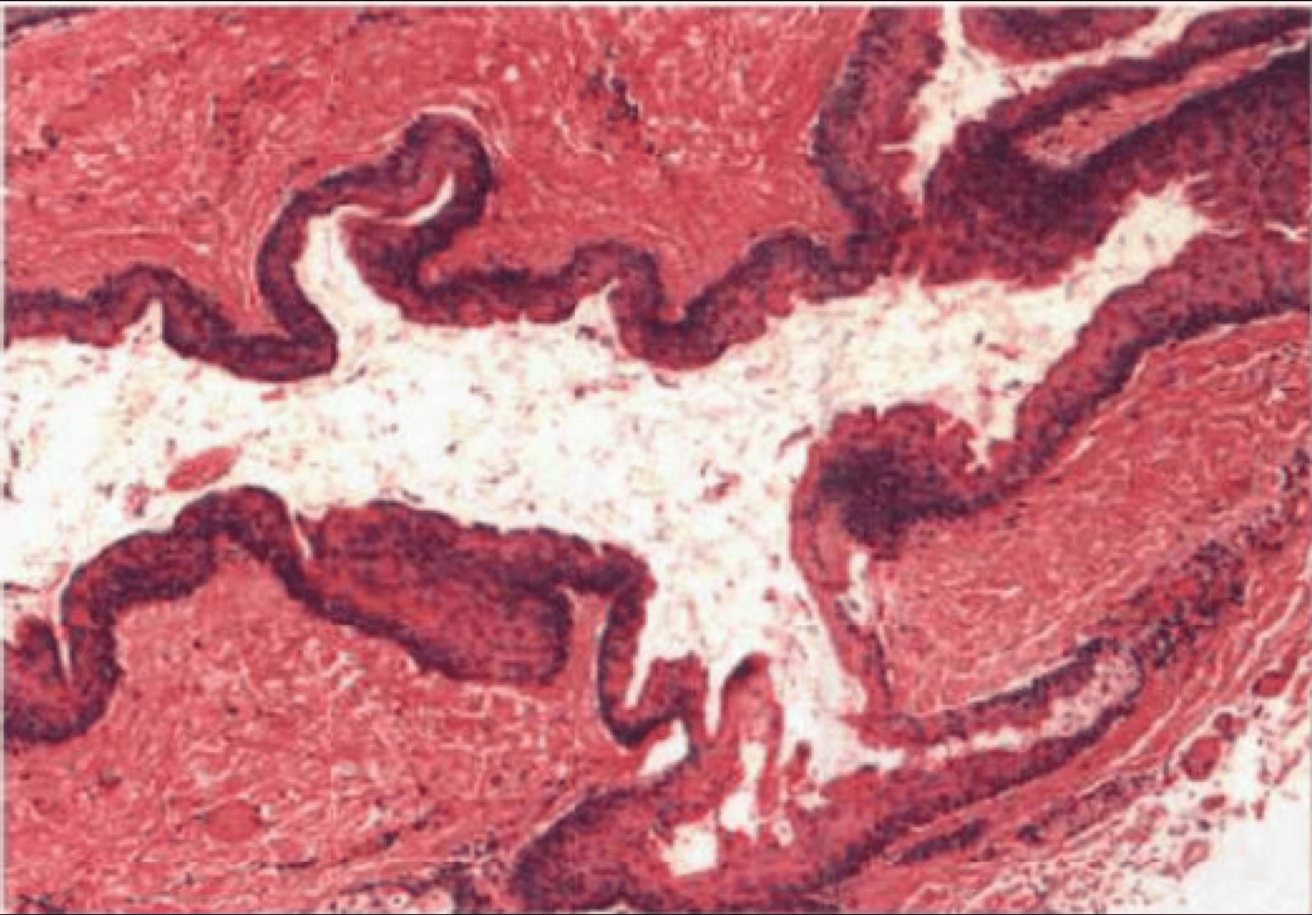
Histopathology of the excised cyst demonstrating the epithelial-lined cyst with sebaceous lobules, thin epithelial lining, and an eosinophilic cuticle, consistent with steatocystoma simplex. This is an original histological image obtained from LabCorp of the excision of the left eyelid of the patient described in this case report.

Microscopic examination revealed an epithelial-lined cyst with sebaceous lobules, thin epithelial lining, and an eosinophilic cuticle consistent with SS (Figure 3).

## Discussion

Steatocystomas are rare orbital lesions that can closely mimic more common pathologies, such as dermoid cysts, often leading to misdiagnosis before histopathological evaluation. This case describes the third published pediatric case and one of the youngest reported presentations of orbital SS and adds to the limited literature describing this pathology in pediatric patients.

The first case described a two-year-old girl with no family history of steatocystomas who presented with a mobile, compressible, subcutaneous mass near the lateral border of her outer left eyebrow [4]. Like our patient, this patient was initially diagnosed with a dermoid cyst based on physical examination and imaging, but surgical removal and histological examination of the lesion revealed a stratified squamous epithelial-lined wall with scattered sebaceous glands, a finding consistent with SS, though the eosinophilic cuticle was not observed. The second pediatric case occurred in a four-year-old girl with no family history of steatocystoma who presented with a 15-month history of a soft, freely movable, non-tender subcutaneous mass in her left eyebrow [5]. She was initially diagnosed with a sebaceous cyst and was treated unsuccessfully with two surgical drainages and antibiotic therapy before excision. Histological analysis disclosed a diagnosis of steatocystoma. At the five-year follow-up, no recurrence was observed.

Across all three pediatric cases, there were several commonalities, including asymptomatic periocular swelling, lack of family history, misdiagnosis before surgical excision and histology, and cure by excision. Our case study is now the second to present SS appearing like a dermoid cyst in a pediatric patient and highlights the importance of keeping SS on the differential for dermoid cyst.

The temporal association with minor periocular trauma in this case raises the possibility that trauma may be a precipitating factor in cyst formation; however, this association has primarily been described with epithelial inclusion cysts, not steatocystomas [11,12].

## Conclusions

SS of the orbit is a rare diagnosis that poses a significant diagnostic challenge, particularly in the pediatric population. With only two previously reported pediatric cases in the literature, the natural history, recurrence risk, and long-term outcomes of orbital SS in children remain poorly understood. 

This case reinforces that clinical and imaging findings are insufficient to definitively distinguish SS from other periorbital cystic lesions. Histopathological evaluation remains the gold standard for diagnosis, and complete surgical excision is the definitive treatment, with no recurrence observed in any of the pediatric cases at follow-up.

The presentation of SS in patients under two years of age across multiple reported cases supports the possibility of a congenital origin, which has meaningful implications for family counseling and surgical planning. Increased recognition of SS as a viable differential for pediatric orbital masses is essential to avoid misclassification and guide effective management.
